# Mutations in HPV18 E1^E4 Impact Virus Capsid Assembly, Infectivity Competence, and Maturation

**DOI:** 10.3390/v9120385

**Published:** 2017-12-19

**Authors:** Jennifer Biryukov, Jocelyn C. Myers, Margaret E. McLaughlin-Drubin, Heather M. Griffin, Janice Milici, John Doorbar, Craig Meyers

**Affiliations:** 1 Department of Microbiology and Immunology, The Pennsylvania State University College of Medicine, Hershey, PA 17033, USA; jenbiryukov@gmail.com (J.B.); jcmyers81@gmail.com (J.C.M.); mdrubin@rics.bwh.harvard.edu (M.E.M.-D.); jem10@psu.edu (J.M.); 2 Department of Medicine, Brigham and Women’s Hospital, Boston, MA 02215, USA; 3 Department of Pathology, University of Cambridge, Cambridge CB2 1QP, UK; hgriffi@nimr.mrc.ac.uk (H.M.G.); jd121@cam.ac.uk (J.D.)

**Keywords:** Human Papillomavirus (HPV), HPV18, infection, viral titer, virion maturation, E1^E4

## Abstract

The most highly expressed protein during the productive phase of the human papillomavirus (HPV) life cycle is E1^E4. Its full role during infection remains to be established. HPV E1^E4 is expressed during both the early and late stages of the virus life cycle and contributes to viral genome amplification. In an attempt to further outline the functions of E1^E4, and determine whether it plays a role in viral capsid assembly and viral infectivity, we examined wild-type E1^E4 as well as four E1^E4 truncation mutants. Our study revealed that HPV18 genomes containing the shortest truncated form of E1^E4, the 17/18 mutant, produced viral titers that were similar to wild-type virus and significantly higher compared to virions containing the three longer E1^E4 mutants. Additionally, the infectivity of virus containing the shortest E1^E4 mutation was equivalent to wild-type and significantly higher than the other three mutants. In contrast, infectivity was completely abrogated for virus containing the longer E1^E4 mutants, regardless of virion maturity. Taken together, our results indicate for the first time that HPV18 E1^E4 impacts capsid assembly and viral infectivity as well as virus maturation.

## 1. Introduction

Human papillomaviruses (HPVs) are small DNA tumor viruses that have been identified as the etiological agent of cervical cancer [1,2]. HPVs are also associated with other anogenital and oral cancers [3,4,5]. To date, over 150 types have been identified. All HPV types infect and replicate exclusively in epithelium, and are subdivided based on their ability to infect either cutaneous or mucosal keratinocytes. HPVs that infect mucosal keratinocytes are further subdivided into low-risk and high-risk types. Low-risk types are associated with benign lesions such as condylomas and warts, with high-risk types causing malignant neoplasms such as cervical cancer [6]. High-risk types 16 and 18 are the most prominent causes of cervical cancer, accounting for around 70% of all cases worldwide [7,8,9].

The HPV genome is circular, double stranded, and approximately 8 kb in length. There are 8 open reading frames, which are expressed from polycistronic messages from two promoters [10,11,12]. Early transcripts, which encode the viral proteins E1, E2, E1^E4, E5, E6, and E7 are expressed from the early promoter, but can become elevated late in infection during genome amplification [10,13]. The E1 and E2 proteins are involved in DNA replication and the regulation of transcription [14,15,16,17,18]. Among the high-risk HPV types, E6 and E7 function as oncoproteins, and bind to p53 and retinoblastoma (Rb) respectively to modulate their function [15,19,20,21,22]. As basal cells divide to produce daughter cells that subsequently differentiate, transcripts initiated at the late promoter facilitate elevated expression of the E1^E4 and E5 proteins, as well as the viral capsid proteins, L1 and L2. New particles are then assembled in the upper layers and progeny virions are released as the cornified cells break down and slough off [13,23,24,25,26,27,28].

The E1^E4 transcript is a product of RNA splicing and contains the first five codons of E1 fused to the entire E4 open reading frame [29,30,31,32,33,34]. The E1^E4 open reading frame is present in most early and late transcripts and the protein is present throughout much of the tissue. However, high levels of protein expression coincide with viral genome amplification in the upper layers of the epithelium [35,36,37]. E1^E4 has been extensively studied and multiple functions of E1^E4 have been elucidated, including an interaction with keratin intermediate filament networks to facilitate network re-organization [28,38,39]. Although their significance is less well defined, E1^E4 proteins can also associate with mitochondria to induce apoptosis [40], bind proteins involved in RNA processing [41], disrupt nuclear dot 10 domains [42], and associate with cellular cyclins to induce cell cycle arrest in the G2/M phase [43,44,45,46], a range of activities that most likely reflect E1^E4 function during the virus life cycle.

The majority of studies identifying biological properties of E1^E4 have been carried out in monolayer cell culture where E1^E4 is over-expressed in poorly differentiating keratinocytes. This is in contrast to the natural expression during the viral life cycle, which occurs mostly in terminally differentiating cells. Therefore, in this study, we investigate the role of HPV18 E1^E4 in the context of the natural life cycle utilizing organotypic raft culture. We compare virus produced with wild-type (WT) E1^E4 with virus produced in the presence of four truncation mutants of E1^E4—17/18, 34, 54, 63/67. Our studies reveal that virus produced in the presence of the longer truncation mutants of E1^E4 (34, 54, 63/67) had significantly lower viral titers and were significantly less infectious compared to wild-type E1^E4. In contrast, the titers and infectivity of the shortest truncation mutant (17/18) was similar to WT. In addition, we analyzed the stability of virus produced from tissue containing the WT and the 17/18 mutant and found that the virus produced in the presence of the 17/18 mutant E1^E4 was much less stable, suggesting that E1^E4 may play a role in virus maturation. Overall, this study indicates for the first time that HPV18 E1^E4 may play a role in viral capsid assembly, viral infectivity, and virus maturation.

## 2. Materials and Methods

### 2.1. Ethics Statement

The use of human foreskin keratinocyte tissues to develop cell lines for these studies was approved by the Institutional Review Board at the Pennsylvania State University College of Medicine (IRB no. 25284NHR; 18 April 2007). Discarded tissues were exempt from needing informed patient consent. Patient identifiers were not attached to any tissue samples and informed consent was waived by both Institutional Review boards.

### 2.2. Generation of HPV18 E1^E4 Mutants

The HPV18 E1^E4 truncation mutants (17/18, 34, 54, and 63/67) utilized in this study were generated via site directed mutagenesis as previously described [47]. Briefly, mutagenesis was carried out using a subgenomic HPV18 genomic fragment that was excised from plasmid pBSSK-HPV18 following digestion with Apa I and cloned into plasmid pBC (Stratagene, Bellingham, WA, USA). This fragment, which contained the HPV18 E4 region, was subsequently used as a template for site-directed mutagenesis using the QuickChange XL Site-Directed mutagenesis system (Stratagene, Bellingham, WA, USA). Mutagenesis primers were typically 39 bases in length with the mutagenized triplet being localized centrally. In all cases, E4 mutations were designed so as to introduce only silent changes into the overlapping E2 open reading frame (ORF). After mutagenesis, the subgenomic fragment was cloned back into pBS-HPV18, and the reconstructed mutant HPV18 genome completely sequenced to confirm that the genome contained only the desired mutations.

### 2.3. Keratinocyte Cultures and Electroporation

Primary human foreskin keratinocytes (HFKs) were isolated and grown from newborn circumcision as previously described [48]. Electroporations were performed by digesting 30 µg of the WT HPV genome or the HPV genomes containing mutant E1^E4 with EcoR I to linearize the viral DNA and separate it from the vector sequence. Primary keratinocytes were electroporated with the prepared DNA as previously described [24,48,49,50]. Immortalized keratinocytes stably maintaining HPV episomes were cultured with J2 3T3 feeder cells (a kind gift from Elaine Fuchs) and maintained in E-medium [24,48,49,50]. At least 3 cell lines were generated for each HPV genome.

### 2.4. Organotypic Raft Culture Derived Native Virion Production

Production of native virions was done by growing immortalized HPV-containing keratinocytes in organotypic raft cultures as previously described [24]. Briefly, cell lines were seeded onto collagen matrices containing rat-tail type 1 collagen and J2 3T3 feeder cells. Following attachment and growth to confluence, the matrices were lifted onto stainless steel support grids and fed, via diffusion from below, with E-medium supplemented with 10 μM 1,2-dioctanoyl-sn-glycerol (C8:O; Sigma Chemical Company, St. Louis, MO, USA). Raft cultures were allowed to stratify and differentiate for a period of 10–20 days to obtain immature and mature virions [51,52].

### 2.5. Histology and Immunofluorescent (IF) Staining of Tissue Sections

Raft tissues were fixed in 10% neutral buffered formalin. Tissue was embedded in paraffin and 4 μM thick sections were cut and stained with either hematoxylin and eosin (H&E) as previously described [24] or assessed via immunofluorescent (IF) staining for the expression of viral L1 or L2 proteins. The detection of E4 and L1 proteins in raft sections was carried out using the pan-specific E4 antibody FH1.1 [53,54] and the HPV16/18 antibody CamVir 1 [55] as described previously [56]. Images were adjusted for brightness and contrast identically. Images are representative of tissue grown from at least 2 different cell lines for each virus type.

### 2.6. Virus Harvest and Isolation

Native virions were harvested from either 10-day or 20-day organotypic raft cultures [51,52]. Virus was isolated by dounce homogenization of tissue in phosphate buffer (0.05 M sodium-phosphate, pH 8.0) as previously described [51]. Post homogenization, rafts were either left untreated to detect total genomes or were treated with MgCl_2_ (final concentration of 2 mM) and 375 U of benzonase for 1 h at 37 °C to removed unprotected genomes. Samples were then adjusted to 1 M NaCl and centrifuged for 10 min at 4 °C and 10,500 rpm to remove cellular debris. Virus preparations were stored at −20 °C for short term and −80 °C for long term.

### 2.7. OptiPrep Purification of Virions

OptiPrep purification was performed as previously described [51,57]. Briefly, OptiPrep solutions were adjusted to 1× Dulbecco’s Phosphate-Buffered Saline (DPBS)/800 mM NaCl. Under-laying was used to create a 27%, 33%, and 39% OptiPrep step gradient. Gradients were allowed to diffuse for 1–2 h at room temperature. Approximately 300 µL of each benzonse-treated virus preparation was layered on top of the gradient and tubes were centrifuged at 234,000× *g* for 3.5 h and 16 °C. After centrifugation, 11 × 500 µL fractions were collected from the top of each tube, with the top fraction being number one and the bottom fraction being number 11, and stored at −80 °C.

### 2.8. Viral Titers

Viral titers were determined as previously described [51,57]. Briefly, viral genomes were released by re-suspension of 10 μL of benzonase-treated virus preparation in 200 μL of Hirt DNA extraction buffer (400 mM NaCl/10 mM Tris-HCl, pH 7.4/10 mM EDTA, pH 8.0), 2 μL 20 mg/mL proteinase K, and 10 μL 10% SDS for 2–4 h at 37 °C. Following extraction, the DNA was purified by adding an equal amount of phenol#x2013;chloroform#x2013;isoamyl alcohol (25:24:1) to the mixture and extracting the aqueous phase. An equal amount of chloroform was added and the aqueous phase extracted again. DNA was then ethanol precipitated overnight at −20 °C. The DNA was pelleted by centrifugation then the pellet washed with 70% ethanol and re-suspended in 20 μL Tris-EDTA (TE). To quantify the viral genomes, a Thermo Scientific Maxima SYBR Green qPCR kit (Waltham, MA, USA) was utilized to amplify the viral E2 ORF. The 5′ primer for amplification of the E2 ORF is 5′-TCCGCTACTCAGCTTGTTAAACA-3′ and the 3′ primer is 5′-CCCACGGACACGGTGC-3′. The silent mutations in the E1^E4 sequence did not affect attachment of the E2 primers. Amplification of the E2 ORF of serially diluted pBSHPV18 DNA, ranging from 10^8^–10^4^ copies/μL served to generate a standard curve. Acceptable *R*^2^ values for the standard curves were at or above 0.95. A Bio-Rad CFX-96 Real-Time qPCR machine (Hercules, CA, USA) and corresponding software were used for data collection and subsequent analysis [57].

### 2.9. Infectivity Assays

HaCaT cells (kindly provided by Norbert Fusenig) were grown in Dulbecco’s Modified Eagle Medium supplemented with 10% (*v*/*v*) fetal bovine serum, 0.11 mg/mL sodium pyruvate, and 0.025 mg/mL gentamicin. For infections, HaCaT cells were seeded 50,000 cells/well in 24-well plates 2 days prior to infection. Virus was mixed with media in a total of 500 μL prior to the addition to cells. A multiplicity of infection (MOI) of 10 was utilized unless otherwise noted. Virus was incubated with the cells for approximately 48 h at 37 °C/5% CO_2_. mRNA was harvested using a Qiagen RNeasy kit (Hilden, Germany). Infections were analyzed as previously described [57]. Briefly, infection was quantified by detection of the HPV18 E1^E4 splice transcript using a RT-qPCR-based assay. Amplifications were performed in duplicate for each sample. Results are representative of means and standard deviation of at least three independent infections for each virus type and at least two different virus preparations for each virus type. Students *t*-test was performed with statistical significance calculated at *p* < 0.05.

### 2.10. Neutralization Assays

HaCaT cells were seeded as described above for the infectivity assay. Prior to infection, virus was incubated with a 1:1000 dilution of anti-L1 H18.J4 (a kind gift from Neil Christensen, The Pennsylvania State University) for 1 h at 37 °C. HaCaT cells were then infected with the virus-antibody mixture and incubated for approximately 48 h. mRNA was extracted and a RT-qPCR assay was used to detect the E1^E4 splice transcript as a measure of infectivity as described above.

### 2.11. SDS-PAGE and L1 Western Blot

The total amount of protein in HPV virus preparations from each sample was quantitated by Bradford Assay. A total of 25 µg of each sample was re-suspended in 6% 2-mercaptoethanol loading buffer and boiled for 10 min. Samples were loaded onto a 7.5% polyacrylamide gel followed by transfer onto a nitrocellulose membrane. Nitrocellulose membranes were blocked overnight using SuperBlock (Thermo Scientific, Waltham, MA, USA) with 0.05% Tween. To detect HPV18 L1, membranes were incubated with H18.7E antibody (1:500). To increase sensitivity, biotin goat anti-mouse IgG (H + L) (1:10,000) (Invitrogen, Carlsbad, CA, USA) was used followed by streptavidin horseradish peroxidase (HRP) conjugate (1:5000) (Invitrogen). Membranes were washed with 1× phosphate buffered saline containing Tween 20 (PBST) after the addition of each antibody. All antibodies were diluted in SuperBlock. HRP was detected using an ECL kit (Perkin Elmer, Waltham, MA, USA).

## 3. Results

### 3.1. Establishment of HPV18 17/18, 34, 54, and 63/67 Cell Lines

**Figure 1:**
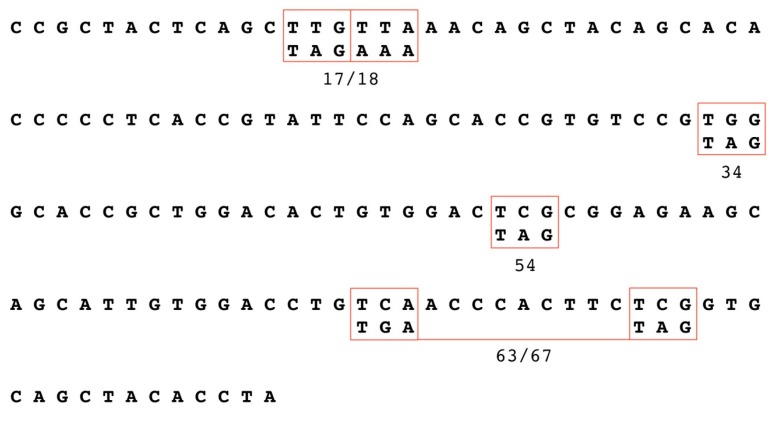
Mutations in the human papillomavirus type-18 (HPV18) E1^E4 protein sequence. Outlined in red are the sites where translation termination stop codons were inserted at amino acids 17/18, 34, 54, and 63/67. The codon modifications are indicated below the wild-type (WT) sequence.

**Figure 2:**
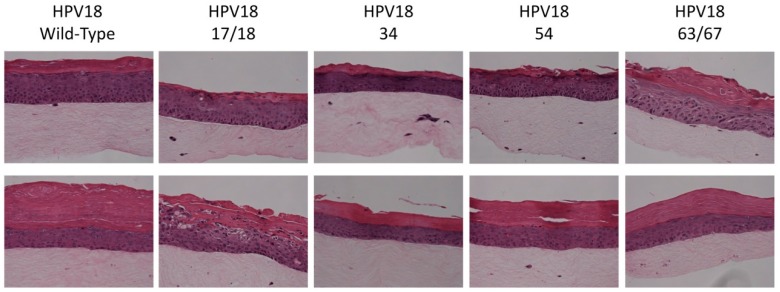
Mutations in the HPV18 E1^E4 protein do not affect organotypic raft growth. HPV18 WT and HPV18 mutant E1^E4 organotypic raft cultures grown for 10 (top row) and 20 (bottom row) days. Tissue sections were cut and stained with hematoxylin and eosin (H&E). Images are 10× magnification. Tissue sections are representative of rafts grown from at least two different cell lines produced for each virus type.

The HPV18 E1^E4 truncation mutants (17/18, 34, 54, and 63/67) utilized in this study are shown in Figure 1. The generation of productive cell lines that produce native HPV virions in fully differentiating epithelial culture were previously described by our lab [24,48,49,50]. We have previously shown that viral maturation occurs in tissue between 10- and 20-days of tissue growth [51]. Therefore, in order to compare mature and immature virions, organotypic raft cultures were harvested at either 10- or 20-days of tissue growth, respectively. Tissue differentiation was similar in organotypic raft tissue sections containing WT or mutant HPV18 genomes at both 10- and 20-days (Figure 2).

### 3.2. Viral Titers Are Reduced in HPV18 Truncation Mutants

**Figure 3:**
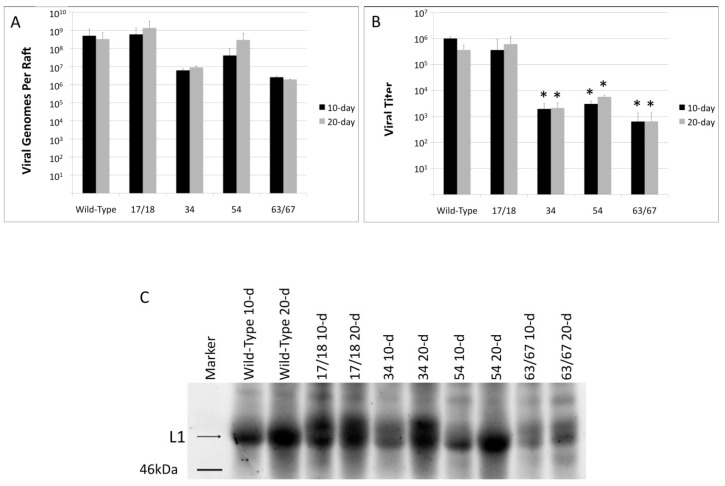

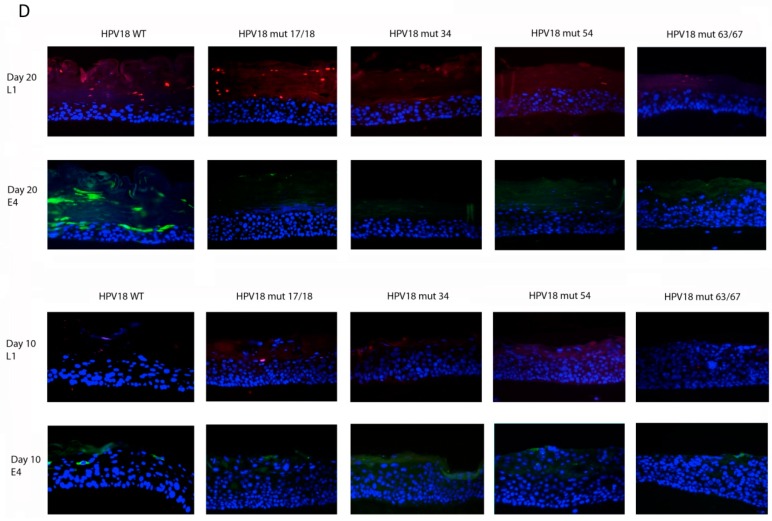
HPV18 virus titer, but not genomic replication or L1 synthesis is affected by mutations in E1^E4. Organotypic rafts harvested at either day 10 or day 20 of tissue growth were homogenized and both (**A**) total viral genomes and (**B**) viral titer were quantified. The total number of viral genomes were quantitated via a qPCR assay amplifying the E2 open reading frame and were measured against a standard curve of known concentrations of HPV genomes. To measure viral titer, homogenized samples were treated with benzonase to remove any non-protected HPV genomes. The quantity of protected viral genomes was then analyzed via qPCR against a standard curve of known concentrations of HPV genomes; (**C**) Western blot analysis of HPV18 L1 (equal loading as determined by Bradford Assay) from 10-day and 20-day WT and HPV18 E1^E4 mutant rafts. The blot was probed with the HPV18 L1 specific H18.7E antibody. The arrow points to HPV18 L1; (**D**) Immunofluorescent (IF) staining of 10—(bottom section) and 20—(top section) day raft tissue with HPV18 L1 (top rows, red) and E1^E4 protein (bottom rows, green). IF images are at 10× magnification. (**A**,**B**) Are averages from at least three individual experiments utilizing at least 2 different sets of rafts/virus preparations. Bars represent standard deviation. An asterisk (*) denotes significance by students *t*-test. Statistical significance was defined as *p* ≤ 0.05. (**C**) is representative of at least two different western blots (WBs) with samples from at least 2 different rafts/virus preparations. IF staining is representative of at least two different raft tissues from two different HPV18 WT and mutant cell lines.

The E1^E4 protein is involved in multiple steps of the viral life cycle [58,59]. Previous studies analyzing mutations in the E1^E4 protein have reported effects in viral genomic amplification and late gene expression [35,58,60,61], but did not explore how virus maturation or infectivity were affected. To determine whether truncations within the HPV18 E1^E4 ORF affected these or other aspects of the viral life cycle in the context of differentiating tissue, total viral genomes from 10- and 20-day raft tissues were quantified (Figure 3A). In contrast to what was observed in the previous studies [35,58,60,61], there was no statistically significant difference in total viral genomes between tissue containing the WT HPV18 genome and tissue containing any of the HPV18 truncation mutants, although there was a strong numerical difference. To determine whether there was a difference in viral titer, virus preparations were treated with benzonase and the number of viral genomes present was quantified (Figure 3B). Benzonase has been shown to eliminate endonuclease-susceptible viral genomes, while leaving the capsid-protected genomes available for analysis [51]. In contrast to what was seen for total genomes, the three longest truncation mutants, 34, 54, and 63/67, had significantly reduced viral titers at both 10-days and 20-days of tissue growth compared to either WT or the 17/18 mutant (Figure 3B). This data indicate that while HPV18 E1^E4 has little affect on viral genome amplification in our system, that it may play a role in viral genome encapsidation.

E1^E4 expression in tissue coincides with viral genome amplification and occurs prior to expression of the capsid proteins. A previous study analyzing the effects of HPV18 E1^E4 mutations found that cells containing mutant HPV18 E1^E4 had reduced levels of late protein expression compared to cells with WT HPV18 E1^E4 [61]. To determine whether, in the context of the natural viral life cycle, the mutations in the E1^E4 protein had an effect on late protein expression, we analyzed the expression of the late capsid protein, L1, in the tissue at both 10-days and 20-days for each E1^E4 mutants by Western blot (Figure 3C) and expression of both L1 and E1^E4 by immunofluorescent staining (Figure 3D). In WT tissue, there is an increase in L1 expression between 10-day and 20-day tissue. Each of the mutants followed the same pattern. Interestingly, the mutants appear to have different levels of the two HPV18 L1 size variants compared to WT (Figure 3C). Overall, the mutants had similar levels of L1 expression compared to WT, with the exception of the 63/67 mutant, which had significantly less L1 present at both 10 and 20 days compared to WT. E1^E4 expression was significantly higher in 20-day tissue compared to 10-day tissue and in WT tissue compared to any of the mutant tissues (Figure 3D).

### 3.3. Relative Infectivity Is Negatively Impacted by Mutations in E1^E4

**Figure 4:**
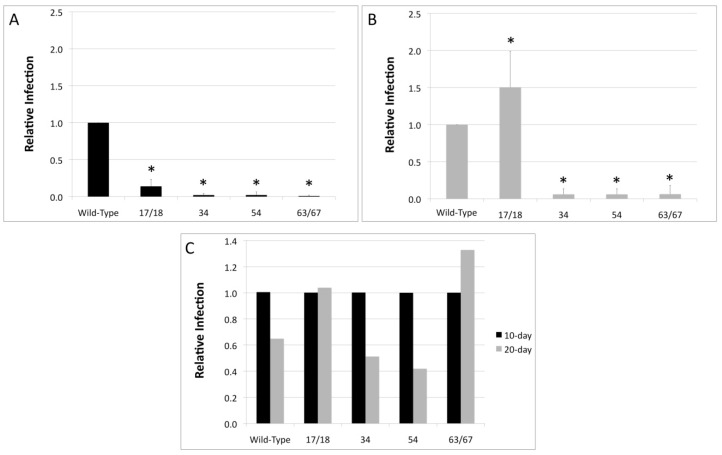
Mutations in HPV18 E1^E4 affect viral infectivity. HaCaT cells were infected with WT or mutant HPV18 virus, harvested at either day 10 or day 20 of tissue growth, at a multiplicity of infection (MOI) of 10. Forty-eight hours post infection (h.p.i.) mRNA was harvested and levels of the E1^E4 splice transcript were determined by RT-qPCR. (**A**) To measure the relative difference in infectivity between 10-day WT HPV18 and the 10-day mutant HPV18 viruses, the level of infection of 10-day WT HPV18 is set equal to one; (**B**) To measure the relative difference in infectivity between 20-day WT HPV18 and the 20-day mutant HPV18 viruses, the level of infection of 20-day WT HPV18 is set equal to one; (**C**) To compare the relative difference between the infection of 10-day virus and 20-day virus for WT HPV18 and each of the HPV18 mutants, each 10-day infection level was set equal to one. (**A**–**C**) Are representative of at least three individual infections utilizing at least two different virus preparations. Bars represent standard deviation. An asterisk (*) denotes significance by students *t*-test. Statistical significance was defined at *p* ≤ 0.05.

Since it was observed that mutations in the E1^E4 protein had an effect on viral titers, we next investigated whether viral infectivity was similarly affected. HaCaT cells were infected with either WT HPV18 or the HPV18 truncation mutants and the E1^E4 splice transcript was amplified in a RT-qPCR-based assay as a measure of infectivity (Figure 4). Relative to 10-day WT virus, the infectivity of the 17/18 mutant was significantly decreased and the infectivity of the 34, 54, and 63/67 mutants was almost completely abrogated (Figure 4A). This was also true for 20-day virus, with the exception of the 17/18 mutant, which had a higher infectivity compared to WT (Figure 4A).

For HPV16, it has been reported that there is a significant difference in the infectivity between 10-day virus and 20-day virus due to maturation of the virus in tissue between 10 and 20 days [51]. In contrast, there is no significant difference in infectivity between 10-day HPV18 virions and 20-day HPV18 virions (Figure 4B). Similarly, there is no significant difference in the infectivity of 10-day versus 20-day virions for any of the HPV18 mutants (Figure 4C).

### 3.4. Mutations in E1^E4 Effect Stability but Not Capsid Conformation

**Figure 5:**
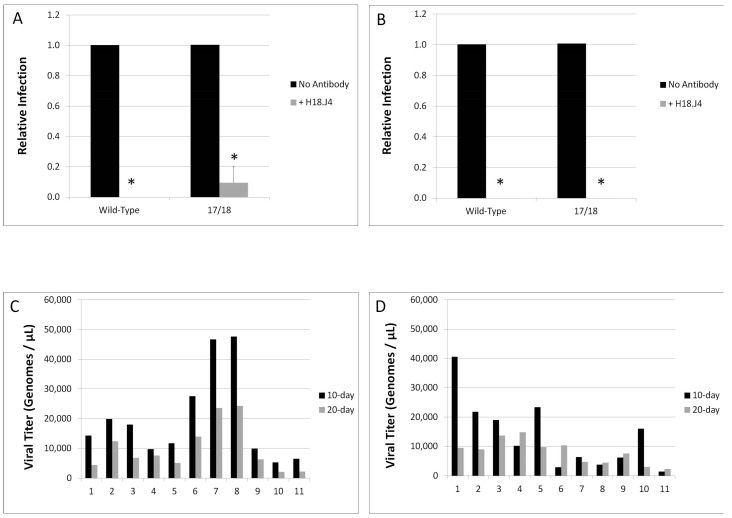
Mutations in HPV18 E1^E4 affects viral stability. HaCaT cells were infected with virus from tissue grown for (**A**) 10 days or (**B**) 20 days. Virus was incubated either alone or in the presence of the HPV18 L1 specific antibody, H18.J4. Forty-eight h.p.i., mRNA was harvested and RT-qPCR was utilized to quantify the E1^E4 splice transcript as a measure of infectivity. Stability of 10-day and 20-day virus for (**A**) WT and (**B**) the HPV18 17/18 E1^E4 mutant was determined by ultracentrifugation. Equal volumes of fractions were used to measure total genome copies per fraction via a qPCR assay amplifying the E2 open reading frame against a standard curve of known HPV18 genome concentrations. (**A**,**B**) Are representative of three individual experiments utilizing at least 2 different virus preparations. The bars represent standard deviation. An asterisk (*) denotes significance by students *t*-test. Statistical significance was defined as *p* ≤ 0.05. (**C**,**D**) Represent fractionation profiles of an individual experiment, with all repeats having a similar trend.

Neutralization of both 10-day and 20-day WT HPV18 and the HPV18 17/18 mutant were tested utilizing the conformation dependent, HPV18-specific L1 antibody, H18.J4. Due to low titers and poor infectivity, we were unable to test the neutralization of the 34, 54, and 63/67 HPV18 viruses. Infectivity of both 10-day and 20-day WT HPV18 was completely abolished in the presence of the antibody (Figure 5A,B). Infection of cells with the 10-day HPV18 E1^E4 mutant was decreased by approximately 90% and infection with the 20-day 17/18 HPV18 E1^E4 mutant was completely abrogated in the presence of the H18.J4 antibody (Figure 5A,B). This is in contrast to what is seen for HPV16, whereby there is a significant difference in the ability of the conformation dependent, HPV16-specific L1 antibody H16.7E to neutralize 10-day versus 20-day HPV16 virions [51]. This data suggests that, while the HPV16 capsid matures between 10-day and 20-day growth in tissue, that HPV18 virions are mature by 10-days of growth in tissue and do not go through additional maturation and conformational changes between 10 and 20-days of tissue growth. 

Previous studies by our lab indicate that the altered infectivity of HPV mutants corresponds to changes in the stability of the viral capsid [51,52,62,63]. Therefore, we investigated whether the HPV18 E1^E4 truncation mutants impacted the stability of the viral capsid. Both 10-day and 20-day WT virions and virions generated with the 17/18 E1^E4 were subjected to OptiPrep gradient ultracentrifugation, separating the mature, stable capsids and the unstable capsids [51,64]. Following ultracentrifugation, fractions were taken and the total genome copies per fraction were quantitated via amplification of the E2 open reading frame in a qPCR-based assay. We have previously reported that fractions 1–4 contain free genomes that have dissociated from immature or unstable capsids and that fractions 6–9 contain mature, intact virions [51]. The fractionation profile of both 10-day and 20-day WT HPV18 can be seen in Figure 5A and indicates that the majority of virus particles are in fractions 6–9, and therefore, stable. In contrast, the fractionation profile of 17/18 HPV18, shown in Figure 5B, indicates that the majority of the HPV DNA is detected in fractions 1–4, or the unstable fractions. This is true for both the 10-day and the 20-day 17/18 HPV18.

In order to compare the stability of WT HPV18 versus 17/18 HPV18, we calculated the virus stability (Table 1). The virus stability is defined as the ratio of total un-protected HPV genomes (fractions 1–4) to the total number of protected HPV genomes (fractions 6–9). Therefore, the lower the number, the more stable the virions are. WT HPV18 virions were highly stable at both 10-days and 20-days, having stability values of 0.470 and 0.457, respectively. In contrast, the 17/18 mutant virus stability changes over time as the virus matures. Virus produced from tissue after 10 days of growth was relatively unstable, with a stability value of 4.76. At 20 days of tissue growth the stability value decreases to 1.76. Presumably this is due to virion maturation between day 10 and 20 of tissue growth. This is comparable to what has been reported for HPV16, whereby immature (10-day) virions were less stable then mature (20-day) virions [51]. This data indicates that E1^E4 may have an impact on the maturation of HPV18 virions in tissue.

## 4. Discussion

The HPV E1^E4 protein has a multitude of functions that contribute to various aspects of the virus life cycle in both high- and low-risk papillomavirus types [58,59]. This study examined whether viral genome amplification, virus assembly, and its infectivity competence was dependent on full-length E1^E4 function. Our data revealed that there was no statistically significant decrease in overall viral genome amplification in any of the raft tissues harboring mutant E1^E4 genomes. This is in contrast to a study utilizing similar truncation mutants in HPV11 E1^E4 where it was observed that the shortest E1^E4 truncation mutant (9 amino acids) actually enhanced viral genome amplification [65]. However, while overall viral genome amplification was not affected, there was a statistically significant effect on virus production. In rafts grown for both 10 and 20 days, tissue containing the three longest HPV18 E1^E4 mutants (34, 54, 63/67) produced significantly lower viral titers compared to tissue containing the HPV18 WT or 17/18 mutant genomes. This result indicates that E1^E4 may play a role in virion synthesis or maturation.

It has previously been demonstrated that mutations in cottontail rabbit papillomavirus (CRPV) E1^E4 resulted in decreased capsid protein expression. In the studying analyzing CRPV E1^E4, the shortest truncation mutant (8 amino acids) of E1^E4 resulted in a decrease in capsid protein synthesis compared to WT or the longer E1^E4 mutants [35]. This is in contrast to our current study where we see a decrease in L1 expression for only the longest truncation mutant (63/67). The 34 and 54 mutants did not have any significant decrease in L1 production compared to WT. Therefore, the decrease in viral titer for the three longer truncation mutants (34, 54, and 63/67) cannot be solely attributed to a decrease in capsid protein expression. These results suggest mutations in the region of the longer truncated mutants may affect capsid assembly and stability. As a result, significant populations of virions are unstable and not efficiently protected by the viral capsid.

Together these studies clearly indicate that E1^E4 is multi-functional and that there are type-specific differences in E1^E4 [58,61,65]. One hypothesis for our finding is the possibility that the longer E1^E4 mutants are misfolded and accumulate in the cell, unable to function in virion formation and capsid stability. In contrast, the 17/18 mutant which is, in fact, often regarded as an “E4 knockout” is too small to be misfolded, and is therefore unable to compromise virus formation and/or stability in the same way. Many viruses produce a large quantity of protein that requires processing, including proper folding, in the endoplasmic reticulum (ER) to support infection. However, here a significant influx of misfolded viral proteins may result in an anti-viral outcome. While it has not been definitively shown, there is evidence that E4 associates with cellular proteins that are necessary for virus assembly and/or maturation [59]. Mutations in the E1^E4 protein may alter the protein structure, preventing binding to or interaction with cellular proteins that are necessary for assembly and/or maturation of virus particles, leading to a decreased viral titer and decreased infectivity from virions produced in cells containing mutant E1^E4 protein. It is possible that immune precipitation of full length, WT E1^E4 would be a potentially effective method to look at cellular binding partners, however, very little protein was detected in rafts containing the mutant E1^E4 proteins. Whether this is owing to a defect in protein production due to the mutations or simply that the antibodies available are unable to detect the mutant proteins is unknown. E1^E4 has previously been shown to interact with the cellular proteins/kinases CyclinA and cyclin-dependant kinase 1 (cdk1) [45,59], however these protein interactions are involved in the process of cellular differentiation early in infection. In this study, there were no differences in raft tissue thickness or any obvious changes in cellular differentiation in tissue containing the mutant E1^E4 proteins. Despite being classified as an early protein, E1^E4 is mainly produced late in infection and it has been shown that L1 and L2 protein are only produced in cells that are already expressing E1^E4, indicating that E1^E4 may play an important role in virion assembly [66]. However, there is very limited information on the interaction with specific cellular proteins late in the viral life cycle. This is presumably due to the complexity of the viral life cycle itself, whereby studies would necessitate differentiating epithelium. While the exact mechanism has yet to be elucidated, we definitively show that mutations in E1^E4 have an effect on the late stages of the viral life cycle, such as capsid assembly and virion stability.

Similar to the decrease in the amount of HPV virions synthesized, we also found a decrease in the infectivity of the virions produced. While there was no overall difference in WT or any of the mutants between 10-day and 20-day virus infectivity, there was a difference in the infectivity between 20-day WT HPV18 and the 17/18 mutant, with the 17/18 mutant having three-fold greater infectivity. We have previously shown that HPV16 native virions mature between days 10 and 20 of tissue growth and that that the infectivity of mature, 20-day virus is significantly enhanced compared to that of immature, 10-day virus [51]. Additionally, maturation of other viruses has been linked to an increase in viral infectivity [67,68]. The 17/18 HPV18 mutant followed a similar pattern of increased maturation and stability over time. This, combined with the poor viral titers for the 34, 54, and 63/67 E1^E4 mutant viruses suggests that E1^E4 may play a role in virion maturation and overall capsid stability. A previous study, utilizing a HPV18 E1^E4 truncation mutant similar to that of the 17/18 mutant used in this study, used Northern blot analysis to show that there was a significant decrease in E1^E4 transcripts in cells containing the mutant genome compared to cells harboring the WT genome [61]. However, the aforementioned study relied on primary HFKs that were transfected with WT or mutant HPV genomes. In contrast, our study analyzed E1^E4 in the context of the complete virus life cycle in organotypic raft culture.

Multiple roles of the HPV E1^E4 protein have been identified in the viral life cycle. This study serves to further elucidate the role of the multi-functional E1^E4 protein, indicating that E1^E4 may play a role in virion maturation and capsid stability.

## Figures and Tables

**Table 1:** Relative stability values of 10- and 20-day wild-type (WT) and human papillomavirus type-18 (HPV18) E1^E4 17/18 mutant virus. Stability values were calculated by dividing the ratio of total genomes in the unstable fractions (fractions 1–4) by the total genomes in the stable fractions (fractions 6–9). The lower the number, the more stable the virions.

Virus Type	Stability
HPV18 Wild-Type 10-day	0.470
HPV18 Wild-Type 20-day	0.457
HPV18 E1^E4 17/18 10-day	4.76
HPV18 E1^E4 17/18 20-day	1.73
